# Harnessing IgM for solid tumor therapy: biology, engineering advances, and translational challenges

**DOI:** 10.3389/fimmu.2025.1712344

**Published:** 2025-11-05

**Authors:** Yuhui Wang, Bing Wang, Shuhan Liu, Yinuo Chen, Shimei Zhang, Lifang Bu, Wenjing Zhu, Xinlin Liu, Peng Sun

**Affiliations:** ^1^ Department of Hepatobiliary and Pancreatic Surgery, The Affiliated Hospital of Qingdao University, Qingdao, China; ^2^ Qingdao Cancer Institute , Qingdao, China; ^3^ Qingdao Medical College, Qingdao University, Qingdao, China; ^4^ Biomedical Center of Qingdao University , Qingdao, China; ^5^ Medical Research Department, Qingdao Hospital, University of Health and Rehabilitation Sciences (Qingdao Municipal Hospital), Qingdao, China

**Keywords:** IgM, immunotherapy, antibody therapy, solid tumor, clinical translations

## Abstract

Immunoglobulin M (IgM) antibodies are gaining renewed attention as next-generation platforms for cancer immunotherapy. Compared with IgG, IgM exhibits distinct biological advantages, including higher avidity from multivalent binding, potent complement activation, and enhanced recognition of heterogeneous tumor antigens within immunosuppressive microenvironments. These attributes position IgM as a promising candidate for solid tumor therapy, despite the absence of currently approved IgM-based therapeutics. Recent advances in genetic engineering, antibody design, and protein manufacturing have enabled the generation of diverse IgM formats—ranging from monoclonal and bispecific constructs to engineered IgM derivatives—demonstrating substantial antitumor potential in preclinical and early translational studies. Nonetheless, clinical development faces persistent challenges, including short serum half-life, restricted tumor penetration, structural and biophysical complexity, and scalability of production. In this review, we discuss the structure and biology of IgM, highlight progress in developing novel IgM-based antibody formats for solid tumors, and critically examine the key translational barriers and future opportunities. Together, these insights underscore the therapeutic promise of IgM and chart a path toward its integration into the next generation of antibody-based cancer immunotherapies.

## 1 Introduction

Immunoglobulins (Igs) are essential glycoproteins that play a central role in the adaptive immune system and are synthesized by B lymphocytes and plasma cells. Humans have five major immunoglobulin isotypes: IgA, IgD, IgE, IgG, and IgM. Each isotype, including its subclasses, exhibits distinct structural and functional characteristics. Among these, IgG is the most abundant serum isotype and has become a cornerstone of cancer therapy due to its unique structural and functional properties (1). IgG antibodies demonstrate high target specificity, thereby enhancing therapeutic safety. Furthermore, IgG mediates immune responses via multiple mechanisms, such as antibody-dependent cellular cytotoxicity (ADCC) and antibody-dependent cellular phagocytosis (ADCP) (1–4). These mechanisms have revolutionized oncology, enabling the development of targeted therapies such as immune checkpoint inhibitors and antibody–drug conjugates (5).

However, IgG therapies face several limitations, including low avidity for antigens with low density or weak affinity as a result of their bivalency (6), restricted penetration into solid tumors, and a limited capacity for potent complement-mediated lysis. These limitations have stimulated interest in alternative isotypes, particularly immunoglobulin M (IgM). IgM antibodies have previously been explored in infectious and autoimmune diseases, where they enhanced pathogen clearance and immune regulation (7). These findings laid the groundwork for their development in cancer. IgM possesses ten binding sites, conferring higher binding avidity than IgG antibodies targeting the same epitope (8). This property enables IgM to bind effectively to low-density or weakly expressed tumor-associated antigens, thereby overcoming a key limitation of IgG. Its pentameric architecture further promotes potent complement activation and direct lysis of tumor cells (9). These functional advantages underscore the potential of developing novel antibody therapies based on IgM. Such therapies may overcome the shortcomings of IgG and provide a promising avenue for the effective treatment of solid tumors (10). In this review, we summarize the structural and biological features of IgM, outline recent advances in IgM-based therapeutic antibodies for solid tumor therapy, discuss major challenges such as short half-life, limited tumor penetration, and manufacturing complexity, and offer perspectives on future directions.

## 2 IgM structure and biology

**Figure 1:**
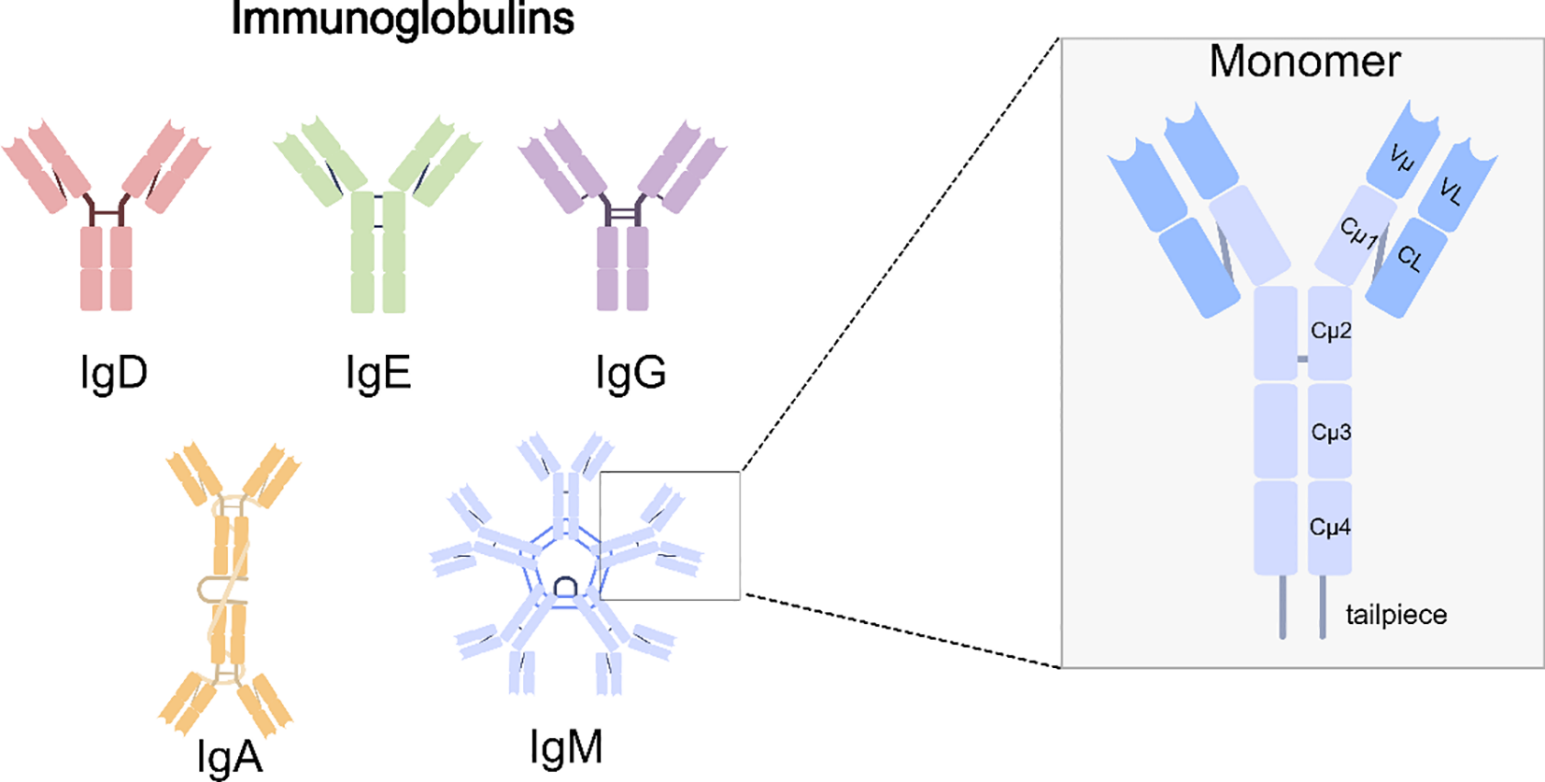
Human immunoglobulin isotypes and IgM structure. Schematic representation of the five major immunoglobulin classes. Among them, IgM is secreted predominantly as a pentamer, conferring ten antigen-binding sites and high avidity. The right panel depicts the IgM monomer, highlighting the variable domains (Vμ, VL), constant domains (Cμ1–Cμ4, CL), and the tailpiece that is essential for multimerization.

Immunoglobulins are proteins produced by immune cells, constituting an essential component of the immune system. They consist of two heavy chains (HCs) and two light chains (LCs). According to the type of heavy chain, immunoglobulins are classified into five isotypes (IgA, IgD, IgE, IgG, and IgM) (11, 12). The heavy and light chains are linked through disulfide bonds to form a Y-shaped structure (13). At the Y-shaped junction, one or more disulfide bonds are typically connected to the heavy chains, forming the hinge region that permits independent movement of the Fab arms and confers relative flexibility between Fab and Fc regions (14). Such a hinge structure is absent in IgM and IgE. The N-terminal region of the immunoglobulin is designated as the variable region and comprises three complementarity-determining regions (CDRs) capable of directly binding antigens, whereas the C-terminal part of the heavy chain is termed the constant region. Most immunoglobulins contain three constant domains (Cμ1–Cμ3), whereas IgM and IgE contain four (Cμ1–Cμ4). Each class is defined by a distinct heavy chain constant region structure that determines its effector functions and biological properties (15). Immunoglobulins are found in plasma and on B-cell surfaces. IgD, IgE, and IgG occur as monomers, while IgA is most commonly present as dimers. IgM exists as a monomer on B-cell surface but polymerizes into either a J chain–containing pentamer or a J chain–independent hexamer, with the pentameric form predominating in humans (16, 17). (An overview of the five immunoglobulin isotypes and the detailed architecture of IgM are presented in **Figure 1**).

IgM is the first antibody isotype generated during the humoral immune response and plays a critical role in mucosal immunity, together with IgA. The IgM light chain comprises ~220 amino acids, whereas the heavy chain consists of ~576 amino acids. The C-terminus of IgM heavy chain contains tailpieces comprising an 18-amino-acid peptide sequence (18). These tailpieces interact with one another, a process essential for IgM polymerization and assembly with the J chain (19). The J chain, a 137-amino acid polypeptide, is an essential component of polymeric IgM and joins two IgM-Fc molecules to stabilize the pentamer. Additionally, it facilitates IgM transport through interaction with polymeric immunoglobulin receptors (pIgR) (20, 21).

Advances in cryo-electron microscopy (cryo-EM) have yielded new insights into IgM structure. Contrary to the previously hypothesized pentagon, single-particle negative-stain electron microscopy revealed that the IgM pentamer adopts an asymmetric pentagon with a pronounced gap (18, 21, 22). High-resolution cryo-EM demonstrated that the pentameric core is an asymmetric, disc-shaped Fc ring formed by the constant regions (Cμ2–Cμ4) of ten μ chains interlaced by disulfide bonds (23). IgM possesses an asymmetric, rigid core formed by the Cμ4 and Cμ3 constant regions and the J chain, with the Fab and Cμ2 domains rotating as a unit around a hinge located at the Cμ3/Cμ2 interface. This structural feature is likely associated with multivalent binding of surface-associated antigens and the activation of the complement pathway (24). The Fc ring is asymmetric and relatively rigid, stabilized by the J chain, whereas the Fab arms exhibit wide mobility in their connection to the Fc ring via the hinge region (24). This architecture enables IgM to bind multiple antigenic epitopes and may facilitate multivalent engagement with tumor-associated antigens (25). Li et al. demonstrated that Fcμ receptor (FcμR) binds specifically to the side of the IgM pentamer rather than in a random manner, and a single IgM pentamer can simultaneously bind up to four FcμR molecules. Moreover, the FcμR binding sites overlap with those of pIgR, suggesting mutually exclusive binding, thereby providing a structural basis for understanding IgM selection in distinct physiological pathways (26). These structural insights further indicate competition between FcμR and pIgR for binding sites, thereby modulating IgM transport and functional pathways (26, 27). Collectively, these observations suggest that IgM exerts potent complement-dependent cytotoxicity (CDC) and may additionally regulate immune balance via receptor-mediated mechanisms.

IgM functions as a critical first line of adaptive immune defense. Its unique structure confers high avidity, enabling efficient pathogen aggregation and toxin neutralization. Early studies showed that IgM activates complement to mediate immune responses (28). More recent studies have revealed that, beyond complement activation, IgM functions through alternative pathways. For example, in solid tumors, IgM may regulate the immune response via non-complement-dependent mechanisms, such as FcμR-mediated pathways (29). Furthermore, although IgM has a larger molecular size than IgG, recent studies indicate that IgM has better relative distribution and selective accumulation in inflamed and tumor tissues due to the extravasation through leaky vasculature and subsequent inflammatory cell-mediated sequestration (ELVIS) phenomenon and the enhanced permeability and retention (EPR) effect (30). These characteristics underscore the promise of IgM antibodies as therapeutic agents in cancer immunotherapy, particularly in the treatment of solid tumors.

## 3 Therapeutic IgM formats in solid tumors

**Figure 2:**
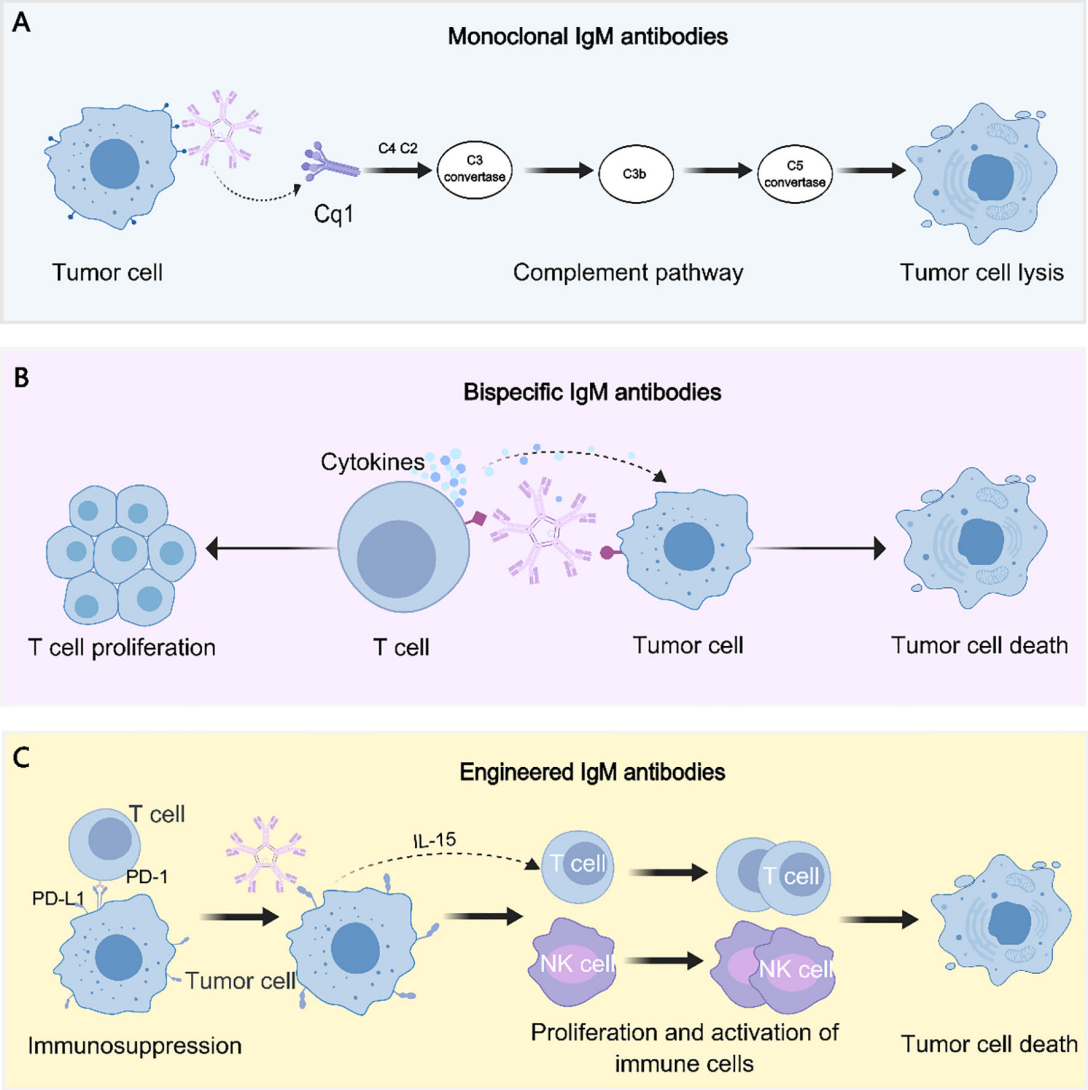
Antitumor mechanisms of IgM antibody formats. **(A)** Monoclonal IgM antibodies mediate tumor cell lysis primarily through potent activation of the classical complement pathway. **(B)** Bispecific IgM antibodies concurrently engage tumor-associated antigens and T cells, thereby promoting cytokine release, immune synapse formation, and tumor cell cytotoxicity. **(C)** Engineered IgM antibodies are designed to overcome immunosuppression (e.g., targeting the PD-1/PD-L1 axis) and to stimulate proliferation and activation of effector immune cells, such as T cells and NK cells, ultimately inducing tumor cell death.

This section outlines the major types of IgM antibodies investigated in tumor therapy and summarizes their current research and clinical status. (Representative antitumor mechanisms of monoclonal, bispecific, and engineered IgM antibodies are presented in **Figure 2**).

### 3.1 Natural IgM

Natural IgM antibodies are primarily secreted by peritoneal B1 B cells and have the capacity to recognize and bind self-antigens. They play critical roles in both immunity and autoimmunity (31). Their polyreactivity and broad specificity enable recognition of pathogen-associated molecular patterns, apoptotic debris, and tumor-associated antigens (31, 32). Mechanistically, natural IgM mediates antitumor activity through two principal pathways. First, it strongly activates the classical complement cascade, inducing CDC and facilitating opsonization of tumor cells (33–35). Second, natural IgM can signal through the FcμR, shaping adaptive immune responses by influencing T- and B-cell cross-talk (36, 37). Together, these mechanisms provide a multifaceted defense against malignant transformation.

Early work demonstrated that IgM antibodies against ganglioside GT1b significantly suppressed Ehrlich solid tumor growth, establishing one of the first links between natural IgM and direct tumor inhibition (38). In breast cancer, natural and adaptive IgM antibodies recognize aberrant glycan structures such as mucins, facilitating immune clearance of transformed cells and preventing tumor progression (39). For example, Atif et al. demonstrated that natural IgM is indispensable for early neoantigen recognition and the activation of adaptive immunity (40). It initiates a cascade of signaling events between monocytes and dendritic cells through immune complex formation, ultimately leading to the activation of CD8^+^ T cells and the induction of cytotoxic responses. This dual role has been validated in two cancer models, urethane-induced tumor and melanoma, underscoring its contribution not only as an innate defense molecule but also as a critical initiator of antitumor immunity (40). These findings suggest novel opportunities for immunotherapy. Natural IgM exhibits strong avidity for repetitive antigens and mediates potent CDC, features that have inspired the design of engineered IgM molecules.

Its unique ability to recognize weakly expressed or structurally altered tumor antigens provides a conceptual foundation for engineering therapeutic IgM molecules inspired by natural prototypes. By leveraging these natural effector mechanisms, engineered IgM antibodies may overcome the limitations of IgG-based antibodies, particularly in targeting heterogeneous and weakly expressed tumor antigens.

### 3.2 Monoclonal IgM antibodies

Monoclonal IgM(mIgM) antibodies are fully human or humanized IgM molecules engineered to bind specific tumor-associated antigens with high affinity. Owing to their multivalent structure, mIgM antibodies can simultaneously engage multiple epitopes with strong avidity. Unlike IgG, which binds only two antigen sites, IgM can effectively target weakly expressed or heterogeneous antigens, making it especially valuable for solid tumor therapy. Recent studies have highlighted unique tumor-killing mechanisms mediated by IgM. In some situations, IgM can induce non-canonical, complement-independent cytotoxicity, including receptor-interacting serine/threonine-protein kinase(RIPK)-independent necroptosis and lipoptosis through lipid accumulation pathways, which are unique pathways that IgG antibodies don’t possess. For example, experimental evidence demonstrated that only IgM antibodies, especially clone M6-1D4, significantly reduce the viability of hepatocellular carcinoma (HCC) cell lines by inducing RIPK-independent necroptosis, while the IgG antibodies were ineffective (41). PAT-SM6 can induce lipoptosis via GRP78–LDL complex internalization (42). These findings emphasize the distinctive advantages of IgM over IgG in solid tumors.

Several mIgM antibodies have shown encouraging preclinical and early clinical potential. IGM-8444 (Aplitabart), although molecularly engineered to enhance DR5 clustering and agonistic signaling, remains a monospecific IgM antibody and is therefore discussed within the monoclonal IgM category (**Table 1**). Preclinical studies revealed that IGM-8444 binds DR5 with high affinity and induces potent cytotoxicity compared with IgG agonists (8). In Colo205 cells, IGM-8444 was more than 10,000-fold more potent than anti-DR5 IgG. Importantly, it exhibited no hepatotoxicity at concentrations up to 500 μg/mL, whereas TNF-related apoptosis-inducing ligand (TRAIL) induced toxicity with an IC_50_ of 0.04 μg/mL. Broad screening across 190 cancer cell lines representing 15 solid and 5 hematological tumors showed strong responses in 25 cell lines (IC_50_ < 2 ng/mL), moderate responses in 75, and weak responses in 90. Combination studies further demonstrated synergistic activity with chemotherapy agents and the BCL-2 inhibitor ABT-199, without additional hepatotoxicity. *In vivo*, IGM-8444 inhibited tumor growth in a dose-dependent manner and achieved complete remission in the gastric PDX model. Collectively, the multivalent structure and efficient cross-linking ability of IGM-8444 address key limitations of IgG-based agonists, providing a promising approach for DR5-targeted therapy.

**Table 1:** Summary of therapeutic IgM antibodies investigated in solid tumors.

Antibody	Institute/Company	Type	Target(s)	MOA	Phase/clinical trial ID/indication	Reference
IGM-8444 (Aplitabart)	IGM Biosciences	Monospecific IgM	DR5	DR5 clustering; apoptosis induction; CDC	Phase 1a/1b/(NCT04553692)/solid tumors	(8)
PAT-SM6	Patrys Ltd.	Monospecific IgM	GRP78 (and GRP78–LDL complex)	Apoptosis; lipoptosis; complement activation	Phase 1 completed/(NCT01727778)/multiple myeloma	(42–46)
SAM-6	Patrys Ltd.	Monospecific IgM	Oxidized LDL receptor variant	Lipid accumulation; lipoptosis	Preclinical/solid tumors	(47, 48)
AT101	Centro Di Riferimento Oncologico (CRO) Di Aviano IRCCS	Monospecific IgM	GPC1	CDC; tumor growth inhibition	Preclinical/solid tumors	(49, 50)
IGM-2323 (Imvotamab)	IGM Biosciences	Bispecific IgM	CD20 × CD3	TDCC; low cytokine release	Phase 1/2/(NCT04082936)/B-cell malignancies	(51, 52)
IGM-2644	IGM Biosciences	Bispecific IgM	CD38 × CD3	CDC; TDCC; low cytokine release	Phase 1/(NCT05908396)/multiple myeloma	(53)
IGM-7354	IGM Biosciences	Engineered IgM	PD-L1 × IL-15	NK/T-cell activation; IL-15 stimulation; antitumor activity	Phase 1 completed/(NCT05702424)/solid tumors	(54)

Another well-studied candidate is PAT-SM6, a human IgM monoclonal antibody targeting a cancer-specific isoform of glucose-regulated protein 78 (GRP78), with additional binding to low-density lipoprotein (LDL) complexes through GRP78-mediated interactions (43) (**Table 1**). GRP78 is aberrantly expressed on the surface of various solid and brain tumors and is implicated in cancer progression (55, 56). PAT-SM6 exerts anticancer activity through apoptosis, proliferation inhibition, CDC, and the unique mechanism termed lipoptosis (42–44). Preclinical studies showed that selective cytotoxicity against melanoma, pancreatic cancer, and multiple myeloma cells while sparing normal tissues. In a Phase 1 trial with 12 heavily pretreated patients with relapsed or refractory multiple myeloma, PAT-SM6 achieved stable disease (SD) in 33.3% of patients, but no partial or complete responses were observed (45, 46, 57). By contrast, SAM-6, another IgM antibody derived from the same research group, specifically recognizes an oxidized LDL receptor variant expressed on malignant cells and induces apoptosis through lipid accumulation (lipoptosis) (47, 48). However, SAM-6 has not yet entered clinical trials; its development remains at the preclinical stage.

Another promising monoclonal antibody is AT101, a complement-fixing mouse IgM that targets glypican-1 (GPC1) (**Table 1**). GPC1 is a cell surface proteoglycan that is highly expressed in pancreatic ductal adenocarcinoma (PDAC) tumor tissues but shows little to no expression in normal pancreatic tissue or chronic pancreatitis (49). It is associated with several growth factors that promote cancer cell proliferation, angiogenesis, and metastasis. AT101 is capable of selectively triggering complement activation and promoting the recruitment of immune effector cells within the tumor microenvironment (TME). In an experiment, it was proven that AT101 can effectively inhibit tumor growth and prolong survival in PDAC xenograft models (50). The data indicate that the average survival time of mice in the AT101 group was significantly longer than that of the control group. Among the mice treated with AT101, most had a reduction in tumor mass, and one achieved complete tumor remission. Moreover, no toxicity was observed in the mice that received multiple injections of AT101. However, AT101 remains in preclinical development, and the critical step in clinical translation will be humanization of the antibody.

Despite these advances, major challenges remain for monoclonal IgM development. Their large molecular size(900–950 kDa for pentamers and 1050–1150 kDa for hexamers), limited stability, and short pharmacokinetic half-life complicates large-scale production and purification (58). Furthermore, most mIgM-based therapies are still in preclinical or early clinical stages, and further optimization, including combination strategies, will be essential to realize their full therapeutic potential.

### 3.3 Bispecific IgM antibodies

The treatment of solid tumors remains highly challenging because of the complexity of the immunosuppressive tumor microenvironment, the heterogeneity of antigen expression, and the limited penetration of large-molecule antibodies into tumor tissues. While monoclonal antibodies provide clinical benefit, their effectiveness is often constrained under those conditions. Bispecific antibodies (bsAbs) have emerged as a representative innovative therapeutic strategy (59, 60). In the bispecific antibodies for treating solid tumors, IgG plays a dominant role due to its longer half-life and efficient immune function. However, their bivalency and limited Fc-mediated clustering often constrain activity in low-antigen-density tumors, motivating the exploration of multivalent alternatives such as IgM (61). Recently, the development of bispecific IgM (bsIgM) antibodies has attracted growing attention, extending beyond infectious diseases to cancer therapy. Although research is still in its early stages, the structural and functional properties of bsIgMs make them a promising approach for overcoming the limitations of existing antibody formats. BsIgMs combine the multivalency of IgM, which has ten antigen-binding sites, with the bispecificity function, and can simultaneously bind to tumor antigens and immune cell markers. This dual capacity provides a distinctive platform for solid tumor treatment. Their high avidity enables effective binding to low-density tumor antigens, and the pentameric structure enhances immune effector activation via complement and Fc receptors (9, 16, 62).

Compared with bispecific IgGs, bsIgMs have demonstrated superior biological activity. For instance, IgM-2323 (Imvotamab), a CD20×CD3 bsIgM, displayed 100-fold higher binding activity to CD20 than IgG-based T cell bispecifics, mediated CDC at levels 100-fold greater, and induced highly potent T cell-dependent cytotoxicity (TDCC) (51) (**Table 1**). In a Phase 1/1b clinical trial (NCT04082936) in relapsed or refractory non-Hodgkin lymphoma (R/R NHL), objective responses were observed in 11 of 38 evaluable patients (29%), including 8complete responses (21%). Notably, activity was seen even in heavily pretreated patients, including those who had undergone CAR-T therapy (52). Based on the encouraging results of IGM-2323 (imvotamab), IGM Biosciences developed a novel CD38×CD3 bispecific IgM T cell engager, IGM-2644 (**Table 1**). It has 10 binding sites for human CD38, and a single anti-CD3 scFv fused to the joining (J) chain. Previous clinical studies have already demonstrated that IGM-2644 exhibits dual CDC and TDCC mechanisms and demonstrates activity against daratumumab-resistant tumor cells. In addition, IGM-2644 also demonstrated reduced T cell fratricide compared to bispecific IgGs (53). Currently, IGM-2644 has an ongoing Phase 1 clinical trial (NCT05908396) for relapsed/refractory multiple myeloma. However, despite this encouraging activity, IGM Biosciences announced in January 2025 that it would terminate all cancer-related pipelines following the failure to achieve expected outcomes and difficulties in strategic development. This result underscores the significant translational challenges facing bsIgMs development. Although preclinical studies indicated potent antitumor activity and reduced cytokine release *in vitro* and in murine models, these findings did not translate consistently into clinical efficacy. The experience with IGM-2323 and IGM-2644 highlights the urgent need to design safer and more effective bsIgM formats.

Despite their promise, bsIgMs face multiple challenges related to structure, manufacturing, and translation. The large pentameric structure of IgM complicates protein folding, stability, and purification, resulting in low yields and batch variability (63). Maintaining high affinity at both binding sites adds further complexity to structural design and production. Additionally, IgM antibodies have relatively short half-lives compared with IgG formats (64), and their large size can hinder penetration and distribution within solid tumors, particularly in dense or immune-excluded tissues. Safety concerns, including immunogenicity and the risks of cytokine release, necessitate cautious dose escalation and rigorous clinical monitoring (54, 65).

### 3.4 Engineered IgM formats

Engineered IgM antibodies are designed to overcome the intrinsic limitations of natural IgM by introducing genetic or structural modifications. These engineered formats leverage the multivalency and immune-activating potential of IgM to enhance tumor targeting, particularly for low-density or heterogeneous antigens. Early studies demonstrated that IgM could serve as an efficient drug carrier. For example, methotrexate-conjugated IgM retained full antigen-binding activity and achieved superior antitumor efficacy *in vivo* compared with free drug or non-specific conjugates (66). Similarly, IgM-based radioimmunoconjugates labeled with α-particle emitters show highly potent and antigen-specific cytotoxicity *in vitro* and *in vivo*, with only a few isotopes per cell sufficient to induce growth inhibition (67).

A representative example is IGM-7354, developed by IGM Biosciences (**Table 1**). This antibody binds multiple PD-L1 receptors while simultaneously trans-presenting a single IL15/IL15Rα complex via the j-chain to activate NK and CD8+ T cells both *in vitro* and *in vivo*. Preclinical studies demonstrated that IGM-7354 exhibits high binding avidity, promotes NK and CD8+ T-cell proliferation, and inhibits tumor growth in PD-L1^+^ triple-negative breast cancer models. It also showed potent single-agent activity in xenograft models, enhanced antitumor effects in combination with ADCC-capable antibodies or CAR T cells, and robust immune activation in cynomolgus monkeys. Based on these data, IGM-7354 entered a Phase 1 clinical trial (NCT05702424) for advanced solid tumors (54). Other engineered IgM molecules, such as IGM-8444, further highlight the capacity of multivalent formats to improve death receptor clustering and amplify apoptosis signaling (8).

Beyond immune checkpoint targeting, other engineered IgM formats are being explored. For instance, the IgM-based T-cell engagers have been designed to activate T cells and induce their killing effect on tumor cells through simultaneously targeting tumor antigens and T-cell receptors (38). Compared with traditional IgG-based bispecific antibodies, IgM-based designs may have higher stability and lower immunogenicity, thereby reducing treatment-related adverse reactions. IgM antibodies have long faced challenges in ADC development due to their high molecular weight, polymeric structure, and a large number of glycosylation sites, but the emergence of chemoenzymatic methods has provided a new platform for the development of IgM-ADCs (68). Recent advances include conditionally activated anti-IgM ADCs. The antibody is shielded by an IgM domain and becomes exposed only in the protease-rich TME. This strategy prevented off-target binding to soluble or normal B cell–expressed IgM, while allowing efficient MMAE-mediated cytotoxicity against malignant IgM^+^ lymphoma cells after activation (69). These findings highlight the diverse strategies of engineered IgM, from T-cell engagers to conditionally activated ADCs, underscoring its therapeutic versatility.

Engineered IgM antibodies provide several advantages compared with IgG or other formats. Their multivalency confers high avidity, enabling efficient binding even to targets expressed at low antigen density within the tumor environment. Although engineered IgMs demonstrate improved stability, extended half-life, and enhanced delivery efficiency compared with natural IgM, significant hurdles remain. From a manufacturing perspective, due to the large molecular size and complex quaternary structure of IgM expression, assembly, and purification often lead to low yields and batch variability. Pharmacokinetically, IgM molecules display rapid systemic clearance and limited tissue penetration, creating a need to balance half-life extension with tumor accessibility. In addition, the multivalency of IgM may increase risks of unwanted complement activation, off-target immune responses, or cytokine release, particularly at high doses or in multifunctional constructs. Advances in protein engineering, optimization of bioprocess, and carefully designed clinical trials will be critical to realize the therapeutic potential of engineered IgM antibodies.

## 4 Challenges and perspectives

Immunoglobulin M (IgM) antibodies are re-emerging as a promising therapeutic modality for solid tumors. Although notable advances have been made in IgM research, design, and structural characterization, several unmet needs remain. Importantly, current IgM studies are still at an early stage, and more reliable preclinical models are required to predict and evaluate efficacy, toxicity, and pharmacokinetics before translation into human clinical trials.

One of the most significant challenges is the short half-life of IgM (9). In 1964, Barth et al. reported that the half-life of IgM was 5.1 days, whereas IgG antibodies exhibit a half-life of up to 21 days or longer (70, 71). This discrepancy is largely attributable to the neonatal Fc receptor (FcRn), which binds endogenous IgG, protecting it from lysosomal degradation and recycling it back into circulation (72). IgM, however, does not undergo this protective pathway. Engineering IgM with FcRn-binding domains (73–76), albumin-fusion motifs, or protective approaches such as liposomal encapsulation or PEGylation (77) has shown promise in extending its circulation time.

In addition to advances in antibody engineering, a deeper understanding of Fc receptor (FcR) biology is essential for optimizing IgM-based therapeutics. FcRs are immune receptors that bind to the Fc region of Igs and play central roles in antibody effector functions (78). While IgG primarily exerts its effects through Fc gamma receptors (FcγRs) to mediate cytotoxic and phagocytic responses, IgM interacts mainly with the complement system and FcμR. Extensive research has focused on FcγRs, which display distinct expression patterns across immune effector cells, including macrophages, dendritic cells, NK cells, neutrophils, and B cells, where they regulate ADCC, phagocytosis, and cytokine production (79). Activating receptors such as FcγRI (CD64), FcγRIIA (CD32A), and FcγRIIIA (CD16A) promote immune activation, whereas the inhibitory FcγRIIB (CD32B) counterbalances these signals to maintain immune homeostasis (80). Understanding this bidirectional regulation provides valuable insight into the rational design of IgM-based therapeutic strategies. FcμR specifically binds to the Fc region of pentameric or hexameric IgM with high affinity, modulating B- and T-cell responses and contributing to immune homeostasis (62). However, its precise role in regulating IgM-mediated antitumor immunity remains largely unexplored, representing a critical frontier for the clinical translation of IgM-based therapeutic approaches.

Despite its multivalency and strong binding avidity, IgM’s large molecular size restricts penetration into dense, stromal-rich tumors. Furthermore, TME features such as elevated interstitial fluid pressure, hypoxia, and acidic pH may impair IgM stability and activity (81, 82). While potent complement activation by IgM can induce tumor cell lysis, it may also amplify pro-inflammatory signaling, thereby exacerbating TME dysfunction (83). Future studies are needed to better elucidate the interaction between IgM and TME, which may enable more precise strategies for tumor targeting. Manufacturability and stability represent additional barriers. The structural complexity of IgM complicates large-scale production and reduces biophysical stability during formulation (9). Encouragingly, advances in related fields have brought new opportunities for IgM development. The concept of developability, which has been critical in the optimization of IgG antibodies (84–86), may similarly help identify superior IgM candidates and streamline drug development. In addition, progress in computational technologies is likely to facilitate the discovery of IgM molecules with enhanced biophysical and pharmacological properties (87). Optimizing expression hosts, applying glycoengineering, and employing machine learning–based developability screening could significantly improve IgM yield and formulation stability.

Recent advances in antibody engineering, expression systems, and bioprocess optimization have begun to address these limitations (58). The future success of IgM therapies for solid tumors will depend on continued progress in antibody engineering, translational biology, and clinical development. With deeper insights into IgM biology and the emergence of innovative formats, improved strategies are expected to overcome current challenges, thereby accelerating the translation of IgM-based therapeutics into clinical trials and ultimately providing new hope for patients with solid tumors.
